# Improvement of Diabetes Mellitus Symptoms by Intake of Ninjin'yoeito

**DOI:** 10.3389/fnut.2018.00112

**Published:** 2018-11-27

**Authors:** Shigekuni Hosogi, Masahiro Ohsawa, Ikuo Kato, Atsukazu Kuwahara, Toshio Inui, Akio Inui, Yoshinori Marunaka

**Affiliations:** ^1^Department of Molecular Cell Physiology, Graduate School of Medical Science, Kyoto Prefectural University of Medicine, Kyoto, Japan; ^2^Department of Neuropharmacology, Graduate School of Pharmaceutical Sciences, Nagoya City University, Nagoya, Japan; ^3^Department of Medical Biochemistry, Kobe Pharmaceutical University, Kobe, Japan; ^4^Research Center for Drug Discovery and Pharmaceutical Development Science, Research Organization of Science and Technology, Ritsumeikan University, Kusatsu, Japan; ^5^Saisei Mirai Clinics, Moriguchi, Japan; ^6^Pharmacological Department of Herbal Medicine, Kagoshima University Graduate School of Medical and Dental Sciences, Kagoshima, Japan; ^7^Research Institute for Clinical Physiology, Kyoto Industrial Health Association, Kyoto, Japan

**Keywords:** Ninjin'yoeito, diabetes mellitus, interstitial pH, insulin resistance, serum glucose, streptozotocin (STZ)

## Abstract

Diabetes mellitus is a well-known common disease and one of the most serious social problems in the worldwide. Although various types of drugs are developed, the number of patients suffering from diabetes mellitus is still increasing. Ninjin'yoeito (NYT) is one of formulas used in Japanese traditional herbal medicines for improving various types of metabolic disorders. However, the effect of NYT on diabetes mellitus has not yet been investigated. In the present study, we tried to clarify the action of NYT on the serum glucose level in streptozotocin (STZ)-induced diabetic mice. We found that intake of NYT decreased the serum glucose level and increased insulin sensitivity in STZ-induced diabetic mice. NYT treatment also improved acidification of the interstitial fluid around skeletal muscles found in STZ-induced diabetic mice, while the interstitial fluid acidification has been reported to cause insulin resistance. Furthermore, in the proximal colon of STZ-induced diabetic mice, NYT treatment showed a tendency to increase the expression of sodium-coupled monocarboxylate transporter 1 (SMCT1), which has ability to absorb weak organic acids (pH buffer molecules) resulting in improvement of the interstitial fluid acidification. Based on these observations, the present study suggests that NYT is a useful formula to improve hyperglycemia and insulin resistance via elevation of interstitial fluid pH in diabetes mellitus, which might be caused by increased absorption of pH buffer molecules (SMCT1 substrates, weak organic acids) mediated through possibly elevated SMCT1 expression in the proximal colon.

## Introduction

Diabetes mellitus is a syndrome caused by metabolic disorders, and leads to several complications including persisted hyperglycemia. Insulin resistance is a well-recognized feature of non-insulin-dependent, type 2 diabetes mellitus due to metabolic disorders. However, insulin resistance is also commonly observed in insulin-dependent (type 1) diabetic patients (1, 2). The insulin resistance obviously appears in peripheral tissues that participate in glucose uptake, glycogen synthesis, and glucose oxidation. Preceding hyperglycemia *per se* or glucose toxicity has been postulated to be an important factor causing insulin resistance in type I diabetes mellitus (3).

Our recent studies indicate a role of interstitial fluid pH in control of insulin sensitivity (4–6). Otsuka Long-Evans Tokushima Fatty (OLETF) rats, a model of type 2 diabetes mellitus, show lowered pH of interstitial fluids around the liver and skeletal muscles, and ascites measured by glass pH microelectrodes (4), and also the brain measured by antimony pH electrodes (7). Moreover, lowered extracellular pH conditions attenuate the intracellular insulin signaling in a rat skeletal muscle-derived cell line by decreasing insulin affinity to its receptor (5), while natural products improve insulin resistance by increasing the interstitial fluid pH in several organs (4).

Ninjin'yoeito (NYT) is composed of 12 crude herbs; rehmannia root, japanese angelica root, atractylodes rhizome, poria sclerotium, ginseng, cinnamon bark, polygala root, peony root, citrus unshiu peel, astragalus root, glycyrrhiza, and schisandra fruit. NYT is a formula used in Japanese Kampo traditional herbal medicines for improving various types of symptoms, including fatigue, anemia, anorexia, night sweats, cold limbs, slight fever, chills, persistent cough, malaise, mental disequilibrium, and insomnia. However, the effect of NYT on diabetes mellitus is unknown. On the other hand, Ninjin-to is composed of 4 medicinal herbs, atractylodes rhizome, ginseng, glycyrrhiza, and processed ginger, and has been reported to prevent the progression of diabetes mellitus in non-obese diabetic mice (8). Moreover, glucose intolerance in obese mice is alleviated by astragalus root through improvement of insulin resistance (9). Interestingly, NYT contains atractylodes rhizome, ginseng, and glycyrrhiza, which are components of Ninjin-to preventing the progression of diabetes (8), Furthermore, NYT also contains astragalus root, which improves insulin resistance (9). These observations suggest that NYT would have potential improving disorders in diabetes mellitus.

Taken together, these observations (4–6, 8, 9) lead us to an idea that NYT would improve disorders in diabetes mellitus by elevating the interstitial fluid pH. Therefore, in the present study, we examined the effect of NYT on serum glucose levels, interstitial fluid pH and insulin sensitivity in streptozotocin (STZ)-induced diabetic mice, and assessed the molecular mechanisms of the effect of NYT.

## Materials and methods

### Ethical approval

The procedures and protocols for the experiments performed in the present study were approved by the Committee for Animal Research of Kyoto Prefectural University of Medicine (No.29-584, 2017).

### Animal

Male ICR 4-week-old mice (Shimizu Experimental Animals, Kyoto, Japan) were used in the present study. The body weight of these mice was about 20 g at the beginning of the experiments. They had free access to food and water in an animal room, the temperature of which was maintained at 24 ± 1°C with a 12-h light-dark cycle (light on at 08:00, light off at 20:00). Diabetic conditions were produced in the mice by an injection of STZ (200 mg/kg body weight; i.v.) prepared in 0.1 N citrate buffer at pH 4.5. Mice with serum glucose levels above 400 mg/dL were considered diabetic. The mice were anesthetized by inhalational isoflurane (3%) when blood samples were collected and muscle surface pH (interstitial fluid pH) was measured. After these procedures, the mice were sacrificed by injecting a high-dose pentobarbital sodium (100 mg/kg body weight; ip). Then, the colon and the kidney were collected from the sacrificed mice.

### Drugs

**Figure 1:**
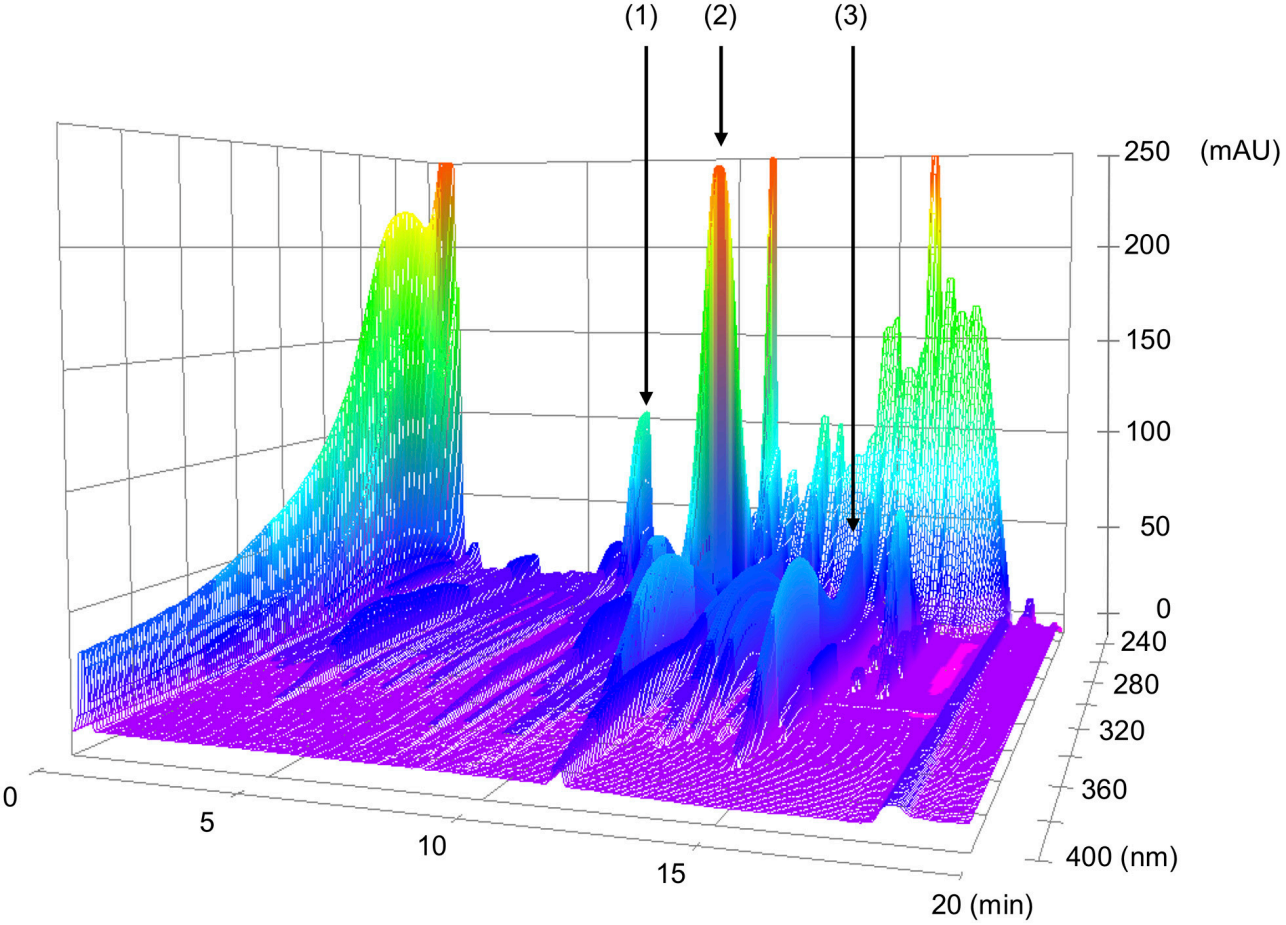
3D-HPLC profiles of Ninjin'yoeito (NYT). Each of chemical marker [paeoniflorin (1), hesperidin (2), or glycyrrhizic acid (3)] in the HPLC profile of NYT was identified by comparison with the retention times and UV spectra (230–400 nm) of their reference standards for quality control.

NYT was gifted from Kracie Pharma, LTD (Tokyo, Japan). NYT of 6.7 g was prepared as a spray-dried powder of hot-water extracts from 12 species of crude drugs: rehmannia root (4.0 g), japanese angelica root (4.0 g), atractylodes rhizome (4.0 g), poria sclerotium (4.0 g), ginseng (3.0 g), cinnamon bark (2.5 g), polygala root (2.0 g), peony root (2.0 g), citrus unshiu peel (2.0 g), astragalus root (1.5 g), glycyrrhiza (1.0 g), and schisandra fruit (1.0 g). A chemical analysis of NYT was performed using the three-dimensional (3D)-high-performance liquid chromatography (HPLC) (Shimazu LCMS-8030 liquid chromatography mass spectrometer equipped with an LC-30AD) with a reversed-phase column (Shim-pack XR-ODSI, 2.0 mm i.d. × 50 mm, 1.6 mm, the column temperature of 40°C) and an SPD-M20A detector with scanning for arrange of 230–400 nm. The procedures were as follows. (1) NYT extract of 0.5 g was mixed and shaken with 50% MeOH of 50 mL, (2) the supernatant of 5 μL was subjected to an HPLC analysis, (3) the column was equilibrated with solvent A (0.1% formic acid in acetonitrile) and solvent B (0.1% formic acid solution in water), and (4) the ratio of solvent A in the mixed solvents, A and B, was initially 5%, elevated by 5% up to 70% over 16 min, and kept at 70% for 1 min with a flow rate at 0.5 mL/min. Since chemical markers, paeoniflorin, hesperidin, and glycyrrhizic acid, are used to keep the quality of NYT as a formula for human patients, the same chemical markers, paeoniflorin, hesperidin, and glycyrrhizic acid, were used to keep the quality of NYT in the present study. The analytical result is shown in Figure 1.

NYT was freshly prepared by mixing in distilled water. Treatment with NYT of oral 1.6 g/kg body weight/day was started from 4 days after the injection of STZ. NYT was resolved in distilled water and all treatments were continued once a day for 3 weeks after STZ administration. Based on the therapeutic dose of NYT for human prescription authorized by Ministry of Health, Labor and Welfare in Japan, the adequate amount of oral NYT intake for mice was determined to be 1.6 g/kg body weight/day [see Tables 11–5 in the report regarding the relationship doses of human and experimental animal^1^].

### Measurement of serum glucose levels and insulin tolerance test

Blood samples were obtained from the tail-vein around 10:00 for determination of casual serum glucose levels under anesthesia with inhalational isoflurane (3%) without any forced fasting. To study insulin tolerance, insulin lispro (Humalog, Eli Lilly, Indianapolis, IN, USA; 0.6 units/kg body weight; i.p.) was administered immediately after the initial blood sample was collected. The blood samples were also collected at 30, 60, 90, and 120 min after insulin administration. Serum glucose levels were determined using glucose CII-test Wako (Wako Pure Chemical Industries, Osaka, Japan). The changes of serum glucose levels were normalized to the glucose level just before the insulin application to make comparisons across the experiment for determination of insulin tolerance.

### Interstitial fluid PH measurement

The interstitial fluid pH around the gastrocnemius muscle was measured under anesthesia with inhalational isoflurane (3%) by attaching the tip of a glass pH microelectrode (Asch Japan, Tokyo, Japan) to the interstitial area around gastrocnemius muscles. The pH measurement was performed just after obtaining the blood sample to determine the casual serum glucose level without any forced fasting 14 days after starting the NYT intake.

### Western blotting of SMCT1

Segments from the proximal colon and the distal colon, and the kidney were removed and homogenized in RIPA buffer containing 50 mM Tris–HCl (pH 7.4), 150 mM NaCl, 0.1% sodium dodecyl sulfate, 0.5% sodium deoxycholate, 1% TritonX, 1 mM phenylmethylsulfonyl fluoride, 25 μg/mL leupeptin, 10 μg/mL aprotinin, 10 mM NaF, and 1 mM Na_3_VO_4_ with dounce tissue grinder method on ice. The homogenate was centrifuged at 15,000 g for 15 min and the supernatant was replaced into fresh tube for further analysis. The protein concentration was determined using BCA protein assay kit (Thermo scientific, Rockford, IL, USA). Proteins were separated on a 10% SDS-PAGE gel and transferred to a nitrocellulose membrane. The membrane was blocked 5% milk in tris-buffered saline for 1 h and then incubated with rabbit anti-SMCT1 antibody (1:5,000) followed by goat anti rabbit IgG-HRP (1:2,000). For the internal control, the same membrane was stripped and re-probed with antibody against ß-actin (1:2,000; Sigma, St. Louis, MO, USA) obtained from Cell Signaling Technology (Beverly, MA, USA). We measured the band densities with Image Lab (BIO-RAD, Hercules, CA, USA) after scanning with Chemidoc XRS Plus system (BIO-RAD).

### Statistical analysis

Data are represented as the means ± standard error (SEM). Statistical significance between the means was assessed by analysis of variance (ANOVA), or Student's *t*-test appropriately. Differences were considered significant at *p* < 0.05.

## Results

### Ninjin'yoeito (NYT) decreased serum glucose levels, but not body weights or amounts of oral food intake

**Figure 2:**
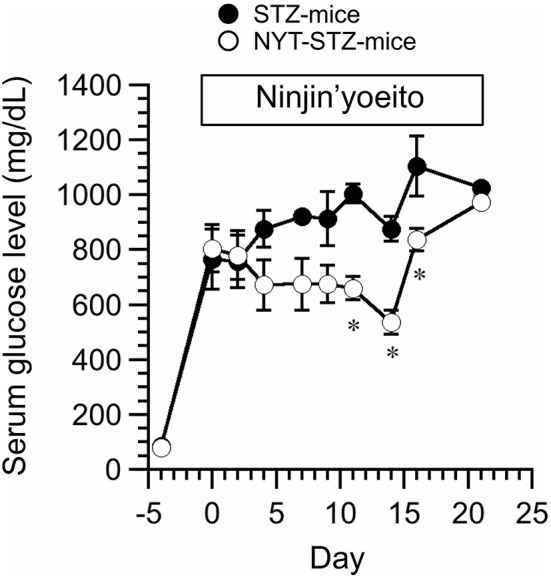
Effects of Ninjin'yoeito (NYT) on casual serum glucose level of STZ-injected mice (STZ-mice) without any forced fasting. STZ was injected 4 days before the NYT administration (day−4 in this figure), and the serum glucose levels of all mice were increased over 400 mg/dL on day 0. NYT administration was started on day 0 and continued up to day 21. STZ-mice without NYT is shown as STZ-mice (closed circles), and STZ-mice with NYT intake is shown as NYT-STZ-mice (open circles). Administration of NYT significantly decreased serum glucose levels (see open circles in this figure; NYT-STZ-mice) at the marked by ^*^p < 0.05, vs. STZ-mice. Each point represents the mean ± SEM of 6 mice.

STZ (200 mg/kg body weight; i.v.) was injected 4 days before application of NYT (Day 0 in Figure 2). We measured the serum glucose level of STZ-injected mice (STZ-mice) with or without NYT (Figure 2). The serum glucose levels just before STZ injection (Day -4 in Figure 2) and 4 days after the STZ injection showed no significant changes (Day 0 in Figure 2) between two groups. The serum glucose levels of STZ-mice became above 400 mg/dL at 4 days after the STZ injection (Day 0 in Figure 2) just before NYT treatment, and all of them were considered diabetes. The serum glucose level of STZ-mice without NYT intake (STZ-mice) continuously increased (closed circles in Figure 2), while the STZ-mice with NYT intake (NYT-STZ-mice) did not show further elevation of serum glucose level within 14 days after starting NYT administration (open circles in Figure 2). The serum glucose levels of NYT-STZ-mice were significantly lower than those of STZ-mice (compare open circles with closed circles on Day 11, 14, and 16 in Figure 2; ^*^p < 0.05). However, 21 days after the starting of NYT intake, the serum glucose level of NYT-STZ-mice (the open circle on Day 21 in Figure 2) was identical to that of STZ-mice without NYT intake (the closed circle on Day 21 in Figure 2).

**Figure 3:**
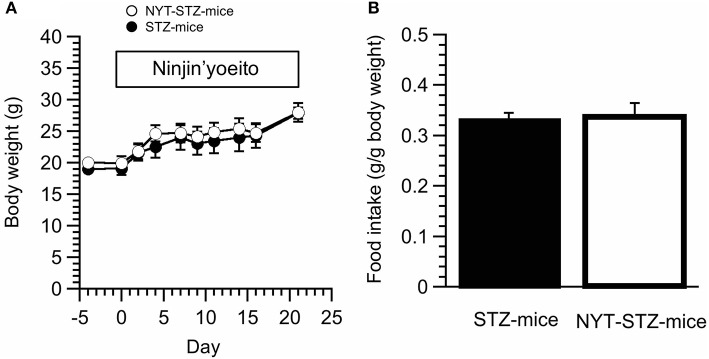
Effects of Ninjin'yoeito (NYT) on body weight and food intake of STZ-mice. **(A)** The body weight was not influenced by NYT intake in STZ-mice. **(B)** The amount of oral food intake was measured 10 days after starting NYT treatment. NYT did not induce any change of oral food intake amounts in STZ-mice. Each point represents the mean ± SEM of 6 mice.

We did not detect any significant changes in the body weight between NYT-STZ- and STZ-mice (Figure 3A). Total food intake was measured at 10 days after starting NYT intake. The NYT intake had no significant effects on the amount of the food intake (Figure 3B).

### NYT improved the decreased PH of interstitial fluid around skeletal muscle in STZ mice

**Figure 4:**
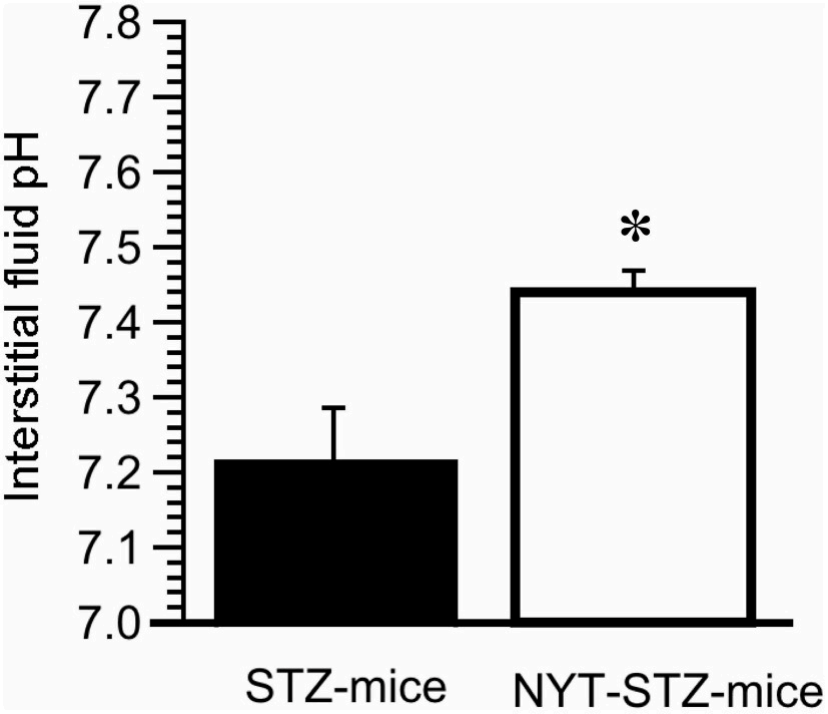
Effects of Ninjin'yoeito (NYT) on the interstitial fluid pH around the gastrocnemius muscle of STZ-mice. The pH measurement was performed just after obtaining the blood sample to determine the casual serum glucose level without any forced fasting 14 days after starting the NYT intake. The interstitial fluid pH around the gastrocnemius muscle was lower in STZ-mice (pH = 7.22 ± 0.07; *n* = 6) compared with that in “non-STZ-injected non-diabetic” mice (pH = 7.40 ± 0.03; *n* = 6; *p* < 0.05). On the other hand, NYT significantly improved the interstitial fluid pH around the gastrocnemius muscle from pH = 7.22 ± 0.07 (the closed column; *n* = 6) to pH = 7.44 ± 0.03 (the open column; *n* = 6; *p* < 0.05), which was identical to that in “non-diabetic” control mice (pH = 7.40 ± 0.03; *n* = 6). ^*^p < 0.05, vs. NYT-untreated STZ-mice group (STZ-mice; the closed column). Each group represents the mean ± SEM of 6 mice.

We have previously reported that the interstitial fluid pH in OLETF rats is significantly lower than normal pH, ~7.40, of control rat (4, 7). Moreover, our recent study indicates that insulin resistance occurs associated with lowered pH of interstitial fluid (5). Therefore, we measured pH of interstitial fluids around gastrocnemius muscles in STZ-mice and NYT-STZ-mice just after obtaining the blood sample to determine the casual serum glucose level without any forced fasting 14 days after starting the NYT intake. The interstitial fluid pH around gastrocnemius muscles of STZ-mice [pH = 7.22 ± 0.07 (*n* = 6)] was lower than that of same-aged “STZ-non-injected” (“non-diabetic”) mice without NYT intake [pH = 7.40 ± 0.03 (*n* = 6); *P* < 0.05]. The NYT intake in STZ-mice significantly elevated the pH value around gastrocnemius muscles from pH = 7.22 ± 0.07 [*n* = 6; the closed column (STZ -mice) in Figure 4] to pH = 7.44 ± 0.03 [*n* = 6; the open column (NYT-STZ–mice) in Figure 4; *P* < 0.05], which was identical to that of “non-diabetic” mice [pH = 7.40 ± 0.03 (*n* = 6)].

### NYT intake improved insulin sensitivity in STZ-mice

**Figure 5:**
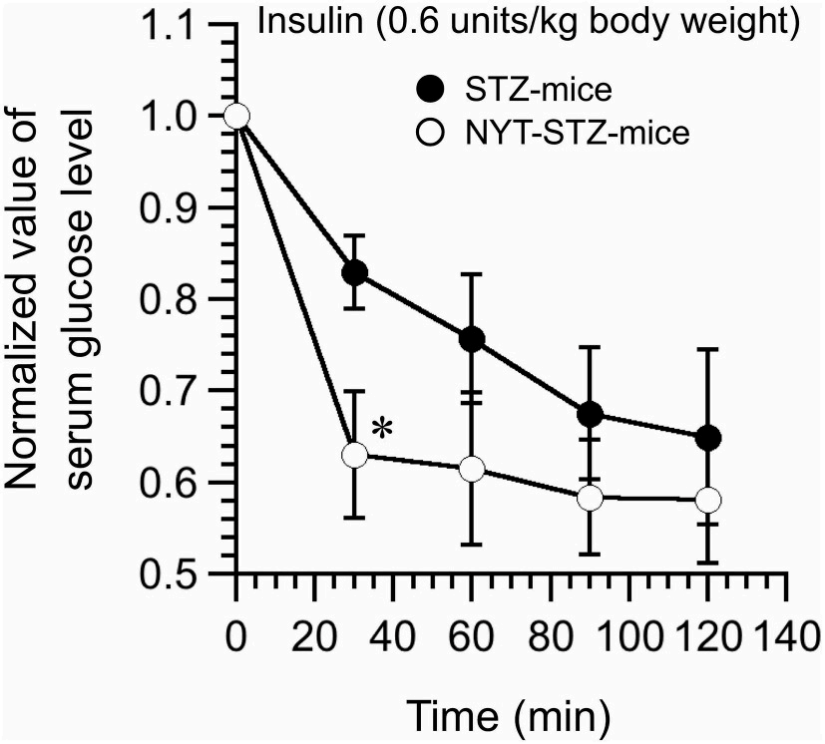
Effects of Ninjin'yoeito (NYT) on the insulin tolerance test of STZ-mice. Insulin (0.6 units/kg body weight; i.p.) was administrated at time 0, and the blood samples were collected at 0 (just before insulin administration), 30, 60, 90, and 120 min after insulin administration. Serum glucose levels faster decreased and reached a steady level in NYT-treated STZ-mice (NYT-STZ-mice; open circles) compared with that in NYT-untreated STZ-mice (STZ-mice; closed circles). ^*^p < 0.05, vs. STZ-mice (the closed circle). Each datum represents the mean ± SEM of 6 mice.

Insulin tolerance test was conducted 16 days after starting the NYT intake. Serum glucose levels of STZ-mice without NYT intake were gradually decreased reaching a steady level at 90 min after insulin administration (closed circles in Figure 5). On the other hand, NYT-STZ-mice showed a faster decrease in serum glucose levels after insulin administration (open circles in Figure 5) reaching a steady level at 30 min after insulin administration compared with that without NYT intake (closed circles in Figure 5). These results suggest that the insulin resistance was improved with NYT intake (Figure 5).

### NYT increased the sodium-coupled monocarboxylate transporter 1 (SMCT1) expression in the proximal colon tissue in mice

**Figure 6:**
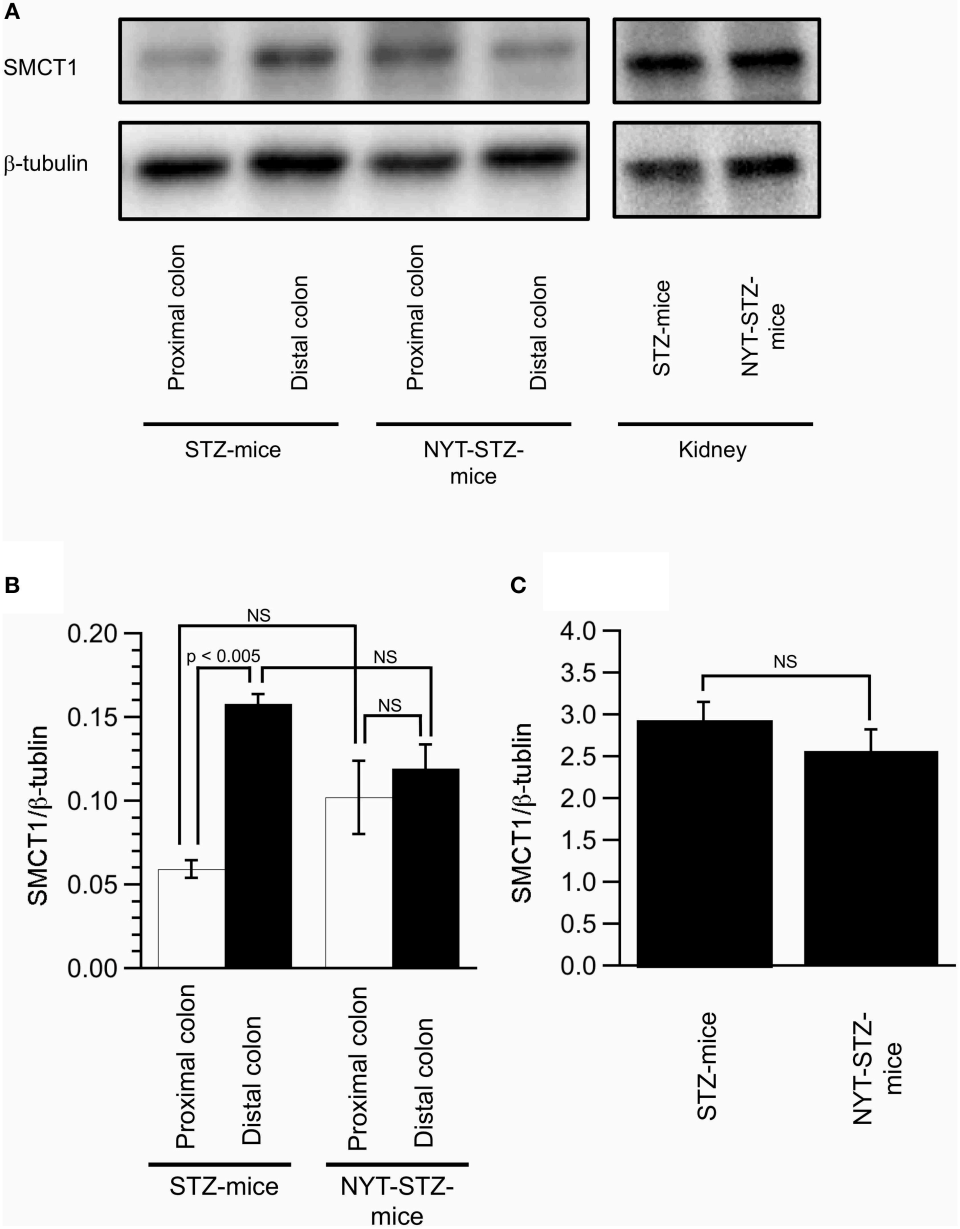
Effects of Ninjin'yoeito (NYT) on the expression of SMCT1 in the colon and the kidney. Images of the expression of SMCT1 protein and ß-tubulin in the proximal and distal colon, and the kidney detected by Western blotting **(A)**. Quantitative results of SMCT1 protein expression were normalized by ß-tubulin measured with Western blotting in the proximal and distal colon **(B)** and the kidney **(C)**. Each datum in **(B,C)** represents the mean ± SEM of 5 mice.

We measured the expression of SMCT1 protein in the colon and the kidney (Figure 6), which transport weak organic acids, mono-carboxyl groups coupled with Na^+^ (10). The SMCT1 expression in the proximal colon of STZ-mice without NYT intake was significantly lower than that in the distal colon of STZ-mice without NYT intake (compare the open column with the closed column in STZ-mice in Figure 6B; *p* < 0.05). The NYT intake showed a tendency to increase the SMCT1 expression level in the proximal colon (compare the open column in STZ-mice with the open column in NYT-STZ-mice in Figure 6B), although we observed no significant effects of NYT intake on the SMCT1 expression in any organs (the proximal or distal colon, or the kidney) measured in the present study (Figure 6).

## Discussion

The present study indicated that NYT diminished the elevated serum glucose levels in STZ-induced diabetic mice (STZ-mice) by improving the lowered interstitial fluid pH in skeletal muscle. The NYT-induced improvement in the lowered pH of interstitial fluids might be due to the increased expression of SMCT1 in the proximal colon based on the observation that the NYT intake showed a tendency to increase the SMCT1 protein expression in the proximal colon.

In the present study, we used STZ-induced diabetic mice as STZ has been applied for production of diabetic conditions. Some compounds have been reported to improve diabetic symptoms (11–14). Empagliflozin, as a selective sodium-glucose co-transporter 2 (SGLT2) inhibitor, improves blood glucose levels, normalizes endothelial function and reduces oxidative stress in aortic vessels of STZ-induced diabetic rats (11). A main component of green tea extract, epigallocatechin gallate, has been reported to preserve insulin secretion to reduce serum glucose levels of STZ-induced diabetic rats (12). These observations indicate that STZ-induced mice are a useful model for diabetic studies.

STZ destroys the islet β cells leading to insulin deficiency, which is applied as a model of type 1 diabetes mellitus. In type 1 diabetes mellitus, the body fluids are acidic due to elevation of lactate and ketone body production (3). Recently, we have reported that the lowered extracellular pH attenuates the insulin-induced intracellular signaling by diminishing the insulin affinity to its receptor in skeletal muscle L6 cell line (5). We have also indicated that organic acids could contribute to the development of insulin resistance in type 2 diabetic animal model OLETF rat (15). In the present study, we indicated that treatment with NYT improved (elevated) the interstitial fluid pH in skeletal muscles, and also improved the insulin-induced diminution of serum glucose levels in STZ-mice. Based on these observations, we propose an idea that NYT lowers serum glucose levels elevated under diabetic conditions by improving insulin resistance via elevation of the lowered body (interstitial) fluid pH, which increases the binding affinity of insulin to its receptor.

Insulin resistance is caused by several factors and events. It is well accepted that enlarged adipose tissue secretes bioactive factors adipokines, such as tumor necrosis factor alpha, interleukin-6, plasminogen activator inhibitor, resistin, and free fatty acid, which attenuate insulin receptor functions (16–18). In case of type 1 diabetes mellitus, visceral fat tropic factors might not be involved in insulin resistance, because adipose tissue in type 1 diabetes is normal or atrophied. It is well accepted that chronic hyperglycemia causes insulin resistance in skeletal muscles (19). Accumulated evidence has indicated the hyperglycemia-induced overproduction of reactive oxygen species (ROS) in the insulin resistance and mitochondrial dysfunction (20). In addition, many epidemiological studies have reported the relationship between metabolic acidosis and insulin resistance (21). Even if the arterial blood pH is strictly regulated within a range between 7.35 and 7.45 even in diabetes mellitus, the observations obtained in the present study indicate that the pH of interstitial fluids around skeletal muscles is at 7.2 in STZ-treated diabetic mice. The activity of various types of enzymes and binding affinity of hormones and neurotransmitters to their receptors are regulated by the pH of interstitial fluids, but not inside blood vessels (6). It is also important to note that the interstitial fluids have little pH buffers unlike blood containing strong pH buffers such as hemoglobin and albumin (6). Therefore, the interstitial fluid pH with little pH buffer in diabetes mellitus would be lower than that under the normal condition, and the lowered pH of the interstitial fluid is a causal factor for the attenuation of insulin signaling in the skeletal muscle.

The studies reported by Goncalves and Martel (22) and Gao et al. (23) would reveal the mechanism of NYT-induced improvement of insulin resistance: SMCT1 transports butyrate (22), and butyrate intake prevents insulin resistance in high-fat diet-fed mice (23). These reports (22, 23) suggest that the elevation of SMCT1 expression would prevent the occurrence of insulin resistance by increasing intake of butyrate. The report (23) indicates that butyrate treatment improves insulin sensitivity by decreasing blood lipids such as triglycerides, cholesterol, and total fatty acids considered as critical factors causing insulin resistance. The present study indicated that NYT treatment improved the interstitial fluid pH around skeletal muscles. The pKa of butyric acid is 4.82. Therefore, under physiological conditions or even under pathophysiological conditions, most of butyric acid (CH_3_-CH_2_-CH_2_-COOH) exists as an ionized form (CH_3_-CH_2_-CH_2_-COO^−^ + H^+^). When butyric acid or butyrate is absorbed across the apical membrane of the intestine, the carboxyl part of butyric acid (CH_3_-CH_2_-CH_2_-COO^−^) is absorbed with Na^+^, but not H^+^ (6). The carboxyl part of butyric acid (CH_3_-CH_2_-CH_2_-COO^−^) plays a role as a pH buffer binding H^+^, resulting in elevation of the interstitial pH (6). Thus, butyrate would show its improving action on insulin resistance via elevation of the interstitial fluid pH through the mechanism as mentioned above. Moreover, butyrate increases mitochondrial respiration, as indicated by the increase in oxygen consumption and carbon dioxide production. Diabetes mellitus shows to reduce mitochondrial function (24–26). Under conditions with ATP production predominantly mediated by glycolysis in impaired mitochondrial function, the total amount of production of H^+^ is much higher than that under conditions with ATP synthesis by glycolysis associated with functional TCA cycle (6). Therefore, it is also possible that butyrate increased in its concentration (content) in the body after NYT treatment would improve mitochondrial respiratory function, resulting in reduction of the production and accumulation of H^+^ in interstitial fluid (elevation of the interstitial fluid pH), which also improves the insulin resistance.

The major active ingredients of NYT responsible for pH homeostasis in the interstitial fluid in diabetic mice are not identified in the present study. Two structurally related natural compounds (astragaloside II and isoastragaloside I) from the medicinal herb astragalus root have been previously reported to improve hyperglycemia, glucose intolerance, and insulin resistance observed in diabetes mellitus by increasing adiponectin secretion in primary adipocytes (9). This pharmacological action of astragalus root seems likely for the mechanisms of NYT-induced improvement of insulin resistance. We have recently reported that daily consumption of propolis that is a natural product derived from plant resins collected by honeybees shows its improving action on insulin resistance by increasing the interstitial fluid pH around the skeletal muscles of OLETF rat (4). Several ingredients in dietary flavonoids are postulated as active components for improvement of diabetic symptoms (27). Although we should try to specify the main active herb contained in NYT showing its improving action on insulin resistance, we should also consider possibility that combined intake of several herbs contained in NYT would be required for NYT to show its improving action on insulin resistance. Further comprehensive studies are necessary to clarify whether any specific ingredient could show the improving action of NYT on insulin resistance or combined intake of several or all ingredients contained in NYT could be required for the NYT action on insulin resistance.

In conclusion, we found that NYT improved the serum glucose levels and insulin resistance in STZ-induced diabetic mice. This improvement by NYT might be due to the alleviation of interstitial fluid acidification though the increased expression of SMCT1 in the proximal colon leading to the absorption of butyrate, pH buffer, via epithelial cells of the proximal colon.

## Author contributions

SH performed experiments and wrote the manuscript. MO performed experiments and wrote the manuscript. IK produced antibodies and contributed to explanation regarding the results. AK wrote a part of the manuscript, contributed to discussion, and explanation regarding the results. TI wrote a part of the manuscript, contributed to discussion, and explanation regarding the results. AI wrote a part of the manuscript, contributed to discussion and explanation regarding the results. YM planned all experimental designs, wrote the manuscript, and contributed to discussion and explanation regarding the results.

### Conflict of interest statement

Kracie Pharma, LTD. (Tokyo, Japan) provided research funds and Ninjin'yoeito, which is a product of Kracie Pharma, LTD. (Tokyo, Japan).

## Footnotes

^1^http://www.zysj.com.cn/lilunshuji/shiyandongwukexue/index.html
